# The relationship between asthma and glioma: a case-control study in a universal access healthcare system

**DOI:** 10.1007/s11060-026-05498-3

**Published:** 2026-03-10

**Authors:** Julie A. Bytnar, Kimberly R. Robins, Brett J. Theeler, Craig D. Shriver, Kangmin Zhu

**Affiliations:** 1 Murtha Cancer Center Research Program, Department of Surgery, Uniformed Services University of the Health Sciences, 6720A Rockledge Drive Suite 310, Bethesda, MD 20817 USA; 2 The Henry M. Jackson Foundation for the Advancement of Military Medicine, Inc, Bethesda, MD USA; 3 Department of Neurology, Uniformed Services University of the Health Sciences, Bethesda, MD USA; 4 Department of Preventive Medicine and Biostatistics, Uniformed Services University of the Health Sciences, Bethesda, MD USA

**Keywords:** Asthma, Glioma, Epidemiology, Case-control study, Protective factors, Military

## Abstract

**Purpose:**

Multiple studies have found an inverse relationship between asthma and malignant gliomas, possibly due to increased immune response from asthma. This study assessed the relationship between asthma and subsequent glioma diagnosis while reducing potential effects of recall and detection bias.

**Methods:**

Data were extracted from the Military Cancer Epidemiology database which includes cancer registry and medical claims data from military and retired servicemembers and their dependents. Cases were individuals with a pathology-confirmed glioma diagnosis between 1998 and 2014. Cancer free-controls were matched to cases by sex, race, age, and active-duty military status. Conditional logistic regression was used to calculate odds ratios (OR) and 95% confidence intervals for the relationship between asthma and subsequent glioma diagnosis.

**Results:**

Cases were less likely to have been diagnosed with asthma than controls [OR = 0.72 (0.57, 0.92)], specifically among high-grade gliomas [OR = 0.71 (0.52, 0.98)] and large (> 37 mm) tumors [OR = 0.60 (0.39, 0.93)]. Cases were less likely to have been diagnosed with asthma 13–36 months [OR = 0.53 (0.33, 0.87)] and > 36 months [OR = 0.75 (0.56, 0.99)] before a glioma diagnosis.

**Conclusion:**

We found an inverse association between asthma and subsequently diagnosed malignant glioma, notably among high-grade gliomas and large tumors. However, no association was observed for small tumors or with asthma diagnosed within 12 months prior to glioma diagnosis, suggesting that greater surveillance after asthma might increase the detection of a glioma. These findings suggest that potential detection bias might have obscured the inverse association between asthma and glioma, particularly among low-grade gliomas and circumscribed gliomas and glioneuronal tumors.

**Supplementary Information:**

The online version contains supplementary material available at 10.1007/s11060-026-05498-3.

## Introduction

An estimated 24,820 cases of malignant brain tumors are expected to be diagnosed in 2025 with over 18,300 deaths resulting from this disease [1]. Malignant gliomas, a type of tumor derived from the brain’s glial cells, are the most common and deadly malignant type of brain cancer [2]. The etiology of malignant gliomas is not clearly understood. Aside from recognized genetic factors, ionizing radiation is one of a few recognized risk factors related to the disease [2].

Asthma is a condition characterized by inflammation and airway hyperactivity. Research has identified an inverse association between asthma and the development of glioma [3, 4]. While the exact mechanisms are unknown, it has been suggested that an increased level of immune response present among those with asthma may play a role [5, 6]. Immunoglobulin E (IgE) is the class antibody that plays a key role in how the body responds to atopic disorders, including asthma. Compared to other classes of immunoglobulins, IgE can exist in tissue for extended amounts of time. As a result, IgE may enhance immune effector cell function by engaging directly with abnormal cells, in turn protecting against development of pre-cancerous cells [7].

There have been epidemiological studies on the relationship between asthma and malignant glioma. A few case-control studies did not find a significant inverse association between asthma and a subsequent malignant glioma; however, these studies had a relatively small sample size without sufficient power to detect an association [8–11]. A case-control study on 4.5 million United States (U.S.) male veterans found an inverse association between asthma of long duration (≥ 2 years) and risk of developing a brain tumor, however this study did not exclusively examine gliomas [12].

Previous studies might be prone to some methodological issues [5, 13]. First, most previous studies used self-reported asthma as the exposure instead of predetermined and more objective criteria for an asthma diagnosis based on medical records [14–20]. Self-reporting may lead to misclassification in assessing asthma exposures, especially for malignant glioma patients for whom cognitive and memory impairment may influence the reporting of asthma history [21]. On the other hand, cases may also report past events and exposures more completely than controls [22]. Such misclassification due to self-reporting might have biased the research results. Second, there might be potential detection bias. Individuals with asthma may have more routine medical follow-ups and are under greater medical surveillance. Thus, they may be diagnosed with malignant glioma earlier than those without asthma, resulting in detection bias. As a result, a true inverse association may appear as no association because of more ascertained asthma among glioma cases than controls.

In order to reduce the effects of recall and detection biases, Kaur et al., conducted a case-control study to address the methodological limitations [21]. To alleviate possible recall bias, medical records were used to confirm an asthma diagnosis. Two sets of matched controls were used to assess and reduce the effects of potential detection bias. The first group consisted of community controls with no history of glioma, and the second group included individuals who underwent a magnetic resonance imaging (MRI) confirming the absence of glioma. They assumed that the MRI-negative controls were more similar to cases in the likelihood of detection of an outcome because they had similar access to healthcare as the cases, due to the use of imaging. Thus, the possibility of detection bias was reduced by using an MRI-negative control group. This study found an inverse association when using MRI-negative controls, but not when using a community control group. However, Kaur et al. had insufficient power to detect a significant association due to the relatively small sample size and lacked generalizability as the sample consisted primarily of White individuals.

The Department of War’s (DoW) Military Health System (MHS) is one of the largest health systems in the U.S [23]. The DoW Central Cancer Registry (CCR) and MHS Data Repository (MDR) contain administrative and medical encounter records for over 9.5 million beneficiaries [24], including active-duty servicemembers, activated reserve servicemembers, military retirees, and their dependents who receive universal healthcare access with little to no out-of-pocket costs [23]. Information on cancer diagnosis and medical history, including asthma, is available in the cancer registry and medical claims data. This case-control study aimed to assess the relationship between asthma and subsequent glioma diagnosis among DoW beneficiaries with universal healthcare access while assessing the potential effects of detection bias on research results by analyzing the data by tumor size and the time interval between asthma diagnosis and a subsequent glioma diagnosis. Additionally, by using health record data rather than self-reports, the possibility of recall bias was reduced for this study.

## Materials and methods

### Data sources and study populations

Data for this study were extracted from the Military Cancer Epidemiology (MilCanEpi) database. MilCanEpi contains demographic, tumor, and treatment data from the DoW CCR linked to administrative and medical encounter data from the MHS MDR for DoW beneficiaries diagnosed with cancer in the MHS as well as MDR records for individuals without cancer records. This database is described elsewhere [25].

In this case-control study, cases were DoW eligible beneficiaries who were diagnosed with a pathology-confirmed malignant glioma between 1998 and 2014. Controls were DoW beneficiaries without any cancer records who were matched to cases by sex, race, age, and military status with a ratio of 4:1, with controls selected to be within ± 1 year of the corresponding case’s age. Controls were assigned an index date corresponding to the date of the glioma diagnosis of the case.

### Study variables

#### Outcome variables

The International Classification of Diseases for Oncology, Third Edition (ICD-O-3) topography (C71, C72) and morphology codes (9380, 9381, 9382, 9383, 9391, 9392, 9393, 9394, 9396, 9380, 9384, 9385, 9400, 9401, 9410, 9411, 9420, 9421, 9423, 9424, 9425, 9430, 9431, 9440, 9441, 9442, 9444, 9445, 9450, 9451, 9460) were used to define glioma [26]. As gliomas have a high degree of heterogeneity, we further categorized tumors into malignant high-grade gliomas, malignant low-grade gliomas, and circumscribed gliomas/glioneuronal tumors using ICD-O-3 morphology codes in conjunction with tumor grade at diagnosis (Supplemental Table A1) [27]. The median glioma size (37 mm) of the study population was used as the cut-point to categorize tumor size as large or small.

#### Exposure variables

A diagnosis of asthma was defined using ICD-9 code 493.x from inpatient or outpatient encounters at any time point prior to the glioma diagnosis (or reference date for controls). The date of the first encounter was used for all analyses.

#### Demographic variables and comorbidities

Demographic variables included sex (male, female), race (White, Black, other, unknown), and age at diagnosis. Military status was categorized as active-duty or non-active-duty. In regard to comorbidities, we calculated the Charlson Comorbidity Index, while excluding asthma and cancer. ICD-9 codes were used to identify medical encounters with diagnosed comorbid conditions (≥ 1 inpatient or ≥ 3 outpatient records for the condition before their cancer diagnosis), other than asthma and cancer, based on the Charlson Comorbidity Index.

### Statistical analysis

The distribution of demographic and clinical characteristics was described by case-control status. The cases and controls were compared in terms of any diagnosis of asthma prior to as well as by the time interval between the first asthma diagnosis and glioma diagnosis (or index date for controls) using conditional logistic regression. The time intervals between the first record of asthma diagnosis and a subsequent glioma diagnosis (or index date for controls) were ≤ 12 months, 13–36 months, and > 36 months. For stratified analyses where there were fewer cases, time was categorized as ≤ 24 months or > 24 months. Analyses were further stratified by tumor size and histologic type. A sensitivity analysis using a more restrictive definition for an asthma diagnosis (at least two outpatient records or one inpatient record) was also performed. All *P*-values were two-sided with a 0.05 significance level. Statistical analyses were conducted using SAS version 9.4 (SAS Institute, Cary, NC) [28].

## Results

There were 1,351 cases of glioma and 5,404 controls in this study. The distributions of demographic and clinical characteristics of the study population are shown in Table 1. The majority of study participants were male (68.5%), White (80.5%), aged 18–64 years (77.4%), and were not active-duty servicemembers (65.5%). The distributions of the matching variables, sex, race, age, and active-duty status at diagnosis, were the same between cases and controls. Controls were more likely to have no comorbidities than cases (84.3% versus 71.7%, *p* < 0.001). Most of the tumors were high-grade gliomas (51.8%). The tumors were approximately evenly distributed between tumors ≤ 37 mm, > 37 mm, and tumors of unknown size.

**Table 1:** Demographic and clinical characteristics, 1998–2014

		Cases	Controls	*P*-value
		*n* = 1,351	*n* = 5,404	
		*n (%)*	*n* (%)	
Sex	Male	926 (68.54)	3,704 (68.54)	1.000
	Female	425 (31.46)	1,700 (31.46)	
Race	White	1,087 (80.46)	4,348 (80.46)	1.000
	Black	119 (8.81)	476 (8.81)	
	Other	108 (7.99)	432 (7.99)	
	Unknown	37 (2.74)	148 (2.74)	
Age	0–17	159 (11.77)	636 (11.77)	1.000
	18–39	513 (37.97)	2,052 (37.97)	
	40–64	532 (39.38)	2,128 (39.38)	
	65+	147 (10.88)	588 (10.88)	
Active-Duty at Diagnosis	Yes	466 (34.49)	1,864 (34.49)	1.000
	No	885 (65.51)	3,540 (65.51)	
Comorbidities	0	969 (71.71)	4,555 (84.29)	< 0.001
	1	239 (17.69)	571 (10.57)	
	2+	143 (10.58)	278 (5.14)	
Pre-existing asthma	No	1,225 (90.67)	4,994 (92.41)	0.034
	Yes	126 (9.33)	410 (7.59)	
Tumor Grade	Low-grade (1/2)	330 (24.43)	--	--
	High-grade (3/4)	694 (51.37)	--	
	Unknown	327 (24.20)	--	
Tumor size	≤ 37 mm	453 (33.53)	--	--
	> 37 mm	442 (32.72)	--	
	Unknown	456 (33.75)	--	
Histologic group	High-grade glioma	700 (51.81)	--	--
	Low-grade glioma	387 (28.65)	--	
	Circumscribed glioma and glioneuronal tumors	264 (19.54)	--	

The proportions of asthma were 9.3% and 7.6% for cases and controls, respectively (*p* = 0.034). However, as shown in Table 2, after controlling for comorbidities, cases were less likely to have been diagnosed with asthma than cancer-free controls (OR = 0.72; 95% CI [0.57, 0.92]). When stratified by histologically defined tumor groupings, this association was statistically significant for high-grade gliomas (OR = 0.71; 95% CI [0.52, 0.98]) but not for the low-grade gliomas (OR = 0.75; 95% CI [0.47, 1.22]) nor circumscribed gliomas and glioneuronal tumors (OR = 0.78; 95% CI [0.42, 1.44]; Table 2). When stratified by tumor size, the association remained for tumors > 37 mm (OR = 0.60; 95% CI [0.39, 0.93]).

**Table 2:** Conditional logistic regression of the likelihood of a glioma diagnosis and a previous asthma diagnosis, 1998–2014

			Cases/Controls	Model 1	Model 2
				OR (95% CI)^a^	OR (95% CI)^b^
All Gliomas	Asthma	no	1,225/4,994	Ref.	Ref.
		yes	126/410	1.27 (1.02, 1.58)	0.72 (0.57, 0.92)
	Comorbidities	none	969/4,555	-	Ref.
		1	239/571	-	2.44 (2.02, 2.95)
		2+	143/278	-	3.67 (2.83, 4.76)
High-Grade Gliomas	Asthma	no	633/2,575	Ref.	Ref.
		yes	67/225	1.22 (0.91, 1.64)	0.71 (0.52, 0.98)
	Comorbidities	none	444/2,226	-	Ref.
		1	145/352	-	2.52 (1.99, 3.20)
		2+	111/222	-	3.72 (2.75, 5.04)
Low-Grade Gliomas	Asthma	no	354/1,443	Ref.	Ref.
		yes	33/105	1.30 (0.85, 1.98)	0.75 (0.47, 1.22)
	Comorbidities	none	306/1,377	-	Ref.
		1	58/132	-	2.43 (1.65, 3.58)
		2+	23/39	-	3.82 (2.07, 7.05)
Circumscribed Gliomas and Glioneuronal Tumors	Asthma	no	238/976	Ref.	Ref.
		yes	26/80	1.36 (0.84, 2.21)	0.78 (0.42, 1.44)
	Comorbidities	none	219/952	-	Ref.
		1	36/87	-	2.10 (1.24, 3.55)
		2+	9/17	-	3.15 (1.21, 8.21)
Small tumor (≤ 37 mm)	Asthma	no	403/1,650	Ref.	Ref.
		yes	50/162	1.29 (0.91, 1.83)	0.75 (0.50, 1.11)
	Comorbidities	none	326/1,512	-	Ref.
		1	84/201	-	2.34 (1.70, 3.22)
		2+	43/99	-	3.04 (1.89, 4.88)
Large tumor (> 37 mm)	Asthma	no	408/1,632	Ref.	Ref.
		yes	34/136	1.00 (0.67, 1.49)	0.60 (0.39, 0.93)
	Comorbidities	none	317/1,467	-	Ref.
		1	77/197	-	2.31 (1.67, 3.20)
		2+	48/104	-	3.14 (2.04, 4.83)
Unknown tumor size	Asthma	no	414/1,712	Ref.	Ref.
		yes	42/112	1.58 (1.08, 2.32)	0.87 (0.56, 1.33)
	Comorbidities	none	326/1,576	-	Ref.
		1	78/173	-	2.64 (1.88, 3.70)
		2+	52/75	-	5.28 (3.33, 8.36)

^a^ Odds Ratio and 95% Confidence Interval: analysis matched for sex, age, race, and military status; ^b^ Odds Ratio and 95% Confidence Interval: analysis matched for sex, age, race, and military status; further adjusted for comorbidities

Table 3 shows that cases were less likely to have been diagnosed with asthma than the cancer-free controls 13–36 months (OR = 0.53; 95% CI [0.33, 0.87]) and > 36 months (OR = 0.75; 95% CI [0.56, 0.99]) prior to a subsequent glioma diagnosis. However, when stratified by histologic group this association was no longer observed for any histologic subgroup (high-grade gliomas: ≤24 months: OR = 0.60; 95% CI [0.34, 1.07]; >24 months: OR = 0.75; 95% CI [0.53, 1.08]; low-grade gliomas: ≤24 months: OR = 0.71; 95% CI [0.25, 2.00]; >24 months: OR = 0.76; 95% CI [0.46, 1.28]; circumscribed gliomas and glioneuronal tumors: ≤24 months: OR = 0.70; 95% CI [0.27, 1.80]; >24 months: OR = 0.81; 95% CI [0.41, 1.61]). Analyses were not stratified by tumor size due to the small percentage of individuals within each category. The results of the sensitivity analyses using a more restrictive definition for a diagnosis of asthma (at least 2 outpatient records or 1 inpatient record) remained similar (data not shown).

**Table 3:** Conditional logistic regression of the likelihood of a glioma diagnosis and the interval between previous asthma diagnosis and glioma diagnosis (or index date), 1998–2014

		Cases/Controls	Model 3
			OR (95% CI)^a^
All Gliomas	Interval ^b^		
	none	1,225/4,994	Ref.
	≤ 12 months	18/49	1.00 (0.57, 1.77)
	13–36 months	23/96	0.53 (0.33, 0.87)
	> 36 months	85/265	0.75 (0.56, 0.99)
	Comorbidities		
	none	969/4,555	Ref.
	1	239/571	2.46 (2.04, 2.97)
	2+	143/278	3.68 (2.83, 4.77)
High-Grade Gliomas	Interval ^b^		
	none	633/2,575	Ref.
	≤ 24 months	16/65	0.60 (0.34, 1.07)
	> 24 months	51/160	0.75 (0.53, 1.08)
	Comorbidities		
	none	444/2,226	Ref.
	1	145/352	2.52 (1.99, 3.21)
	2+	111/222	3.72 (2.74, 5.04)
Low-grade Gliomas	Interval ^b^		
	none	354/1,443	Ref.
	≤ 24 months	5/19	0.71 (0.25, 2.00)
	> 24 months	28/86	0.76 (0.46, 1.28)
	Comorbidities		
	none	306/1,377	Ref.
	1	58/132	2.43 (1.65, 3.58)
	2+	23/39	3.82 (2.07, 7.04)
Circumscribed Gliomas and Glioneuronal Tumors	Interval ^b^		
	none	238/976	Ref.
	≤ 24 months	7/24	0.70 (0.27, 1.80)
	> 24 months	19/56	0.81 (0.41, 1.61)
	Comorbidities		
	none	219/952	Ref.
	1	36/87	2.10 (1.24, 3.55)
	2+	9/17	3.13 (1.20, 8.18)

^a^ Odds Ratio and 95% Confidence Interval: analysis matched for sex, age, race, and military status; further adjusted for comorbidities; ^b^ Time intervals between an asthma diagnosis and a subsequent glioma diagnosis

## Discussion

Using cancer registry and medical claims data, this study found an inverse association between asthma and glioma, specifically among high-grade gliomas and gliomas > 37 mm in size. The inverse association with all gliomas was apparent for asthma diagnosed at least one year prior to a glioma diagnosis, but not within one year. Our findings suggest that asthma may be related to decreased risk of high-grade and large gliomas while potential detection bias may obscure such an association.

Using the cancer registry and medical claims data from a large sample of cases and controls, we found an inverse relationship between asthma and a subsequent glioma diagnosis after adjustment for Charlson Comorbidity Index in addition to other potential confounders. The comorbidity index included medical conditions that may be more common among individuals with severe asthma and may influence detection of glioma, such as chronic obstructive pulmonary disease, cardiovascular disease, and diabetes mellitus [29–32]. Thus, adjustment for the comorbidity index might be important for controlling for the effects of comorbidities that might confound or bias the research results. Our findings of inverse association between asthma and glioma support previous findings [14–21]. The exact mechanism for this apparent protective effect remains elusive, but several have been suggested. Some evidence has implicated histamine levels being inversely associated with immunosuppressive myeloid cells [33]. Another study found that asthma is associated with changes in the function of T cells, which suppresses the ability of microglia to migrate and support glioma growth [34]. IgE levels, which are often deficient in people with cancer, have been identified as a potential novel biomarker for cancer susceptibility [35]. Additional research is needed to identify the mechanisms underlying the inverse association between asthma and risk of developing glioma.

Detection, or surveillance, bias typically inflates estimates of the study outcome due to increased medical surveillance for another medical condition (the study exposure) [36], because patients with the medical condition may seek medical care more frequently and thus are more likely to have tumors detected or diagnosed earlier. Thus, the association between asthma and glioma would be stronger for lower-grade gliomas or tumors of a smaller size. This study found that among individuals with low-grade gliomas, circumscribed gliomas/glioneuronal tumors, and tumors ≤ 37 mm, the inverse association with a prior asthma diagnosis that was present for larger tumors and high-grade gliomas disappeared. These findings are consistent with a previous study which also found a lack of association between asthma and subsequent low-grade gliomas but found an inverse association among high-grade gliomas (OR = 0.62, 95% CI [0.43, 0.88]) [17]. These findings suggest that possible detection bias might have concealed the inverse association which would otherwise have been found for lower-grade or small-sized tumors.

The other approach for assessing possible detection bias is to examine the relationship between asthma and glioma by the time interval between the two. As a result of increased medical care for asthma, an overestimated or false association from detection bias may be more likely to be identified for asthma diagnosed shortly before a glioma diagnosis. We found no association of a glioma diagnosis in the year following an asthma diagnosis, but there was an inverse association with asthma diagnosed more than a year before a glioma. A prior study found no significant association between diagnosed asthma identified via hospital discharge codes and a subsequent brain cancer diagnosis, but they did find an increased relative risk of asthma diagnosed within two years of a glioma diagnosis [12]. These findings suggest that detection bias is likely to be present.

Many previous studies evaluated the relationship between asthma and subsequent glioma used self-reported asthma as the main exposure which may have introduced recall bias into these studies, leading to an overestimated inverse association [8–11, 14–20, 37, 38]. Kaur et al. was able to replicate the inverse association between a confirmed asthma diagnosis through medical record review and a subsequent glioma diagnosis that was found among these previous survey-based studies [21]. This study sought to reduce recall and detection bias by using predetermined criteria for an asthma diagnosis based on medical record review and by using controls from the same community, respectively. However, the power to detect an association was limited due to the relatively small sample of cases and controls. An additional limitation of this study was that gliomas were not grouped based on similar biologic characteristics. Our study adds to the literature supporting the inverse relationship between asthma and subsequent glioma with care taken to reduce recall bias by using diagnosed asthma confirmed through medical claims and to assess the potential effects of detection bias on research results by analyzing the data stratified by tumors of similar biologic composition and time interval from asthma diagnosis and glioma diagnosis.

After stratifying by histologic type, while the adjusted ORs for both ≤ 24 and > 24 months tended to be lower than one for high-grade glioma, the CIs of the adjusted ORs included one (no significant association). Due to smaller numbers of patients after stratification by histology, the time intervals used for this subgroup analysis were broader than the overall analysis and therefore, further research with larger numbers of patients by histology is warranted. Given the highly aggressive and fast-growing nature of high-grade gliomas [39], detection bias may not be likely to play a significant role in any association between their diagnosis and that of a prior asthma condition.

The strengths of our study include a population-based study with a relatively large number of cases and controls from a universal healthcare system and the use of medical claims data rather than self-reports to define the study exposure (asthma). A study on persons with universal healthcare access can reduce the potential influences of differential access to medical care on the identification of asthma. Additionally, we attempted to account for heterogeneity within tumors by grouping histologically and clinically similar tumors together. However, our study also has limitations. Although we used the first record of an asthma diagnosis, the date of diagnosis may not reflect the initial onset of asthma. Asthma was defined based on the availability of data, and thus asthma before the inception of this database was not included, which might have underestimated the frequency of asthma. The time interval between an initial asthma and glioma diagnosis was relatively short in our study, while a latent period for cancer is typically longer. In addition, the time interval of ≤ 24 months was used instead of a shorter period for analysis stratified by histology/grade groupings due to the smaller number of study subjects. To identify the effects of detection bias, a shorter time interval would have been better. Our analyses were limited by the absence of molecular marker data (e.g., IDH mutation, 1p/19q codeletion), which restricted us to classification based on histology and grade; access to molecular information would have allowed for more biologically meaningful tumor categorization. Additionally, the numbers of glioma cases were relatively small for analysis by tumor size. Another limitation is that we did not distinguish specific types of asthma (intrinsic, extrinsic, unspecified) due to the small sample sizes in subgroups. We do not exclude the possible confounding by unmeasured factors such as other atopic conditions or regular use of anti-inflammatory medications or antihistamines. Finally, this study population was DoW beneficiaries. Although most study subjects were not active-duty members, who might therefore be more similar to the general population, findings may not be generalizable to the general population.

This study found a significant inverse association between asthma and subsequently diagnosed gliomas, most notably among high-grade gliomas and large tumors. The lack of an association for low-grade gliomas and small-sized tumors and the period closest to glioma diagnosis suggests that greater surveillance due to asthma might increase the detection of glioma in cases and thus conceal the inverse association between asthma and glioma. Our study provides more evidence on the possibility of reducing the risk of glioma by asthma in the context of considering the potential effects of detection bias.

## Supplementary Information

Below is the link to the electronic supplementary material.

Supplementary Material 1

## Data Availability

The data that support the findings of this study are not publicly available due to the restrictions in the access and use of the MilCanEpi data specified in the data sharing agreements and regulatory approvals. The Department of War Central Cancer Registry (DoW CCR) data and data dictionary may be requested from the Joint Pathology Center and online at https://jpc.capmed.mil/. The Military Health System Data Repository (MDR) data and data dictionary may be requested from the Defense Health Agency and online at https://www.health.mil/.

## Declarations

### Disclaimers

The contents of this publication are the sole responsibility of the authors and do not necessarily reflect the views, opinions, or policies of the Uniformed Services University of the Health Sciences (USUHS), the Henry M. Jackson Foundation for the Advancement of Military Medicine, Inc., the Department of War (DoW) or the Departments of the Army, Navy, or Air Force. Mention of trade names, commercial products, or organizations does not imply endorsement by the U.S. Government.
