# Supplementary material for: The relationship between asthma and glioma: a case-control study in a universal access healthcare system
Source: J Neurooncol. 2026 Mar 10;177(1):38. doi: 10.1007/s11060-026-05498-3 (PMC12975835; doi:10.1007/s11060-026-05498-3)
Supplement: Supplementary file 1 — Supplementary Material 1 [file 11060_2026_5498_MOESM1_ESM.docx]

**Appendix Table A1.** Diagnosis codes and tumor grade used to group gliomas

| **Glioma Category** | **ICD-O-3 Morphology Code** | **Tumor grade(s)** |
| --- | --- | --- |
| High-grade glioma | Anaplastic astrocytoma (9401) | Grade 4 |
|  | Anaplastic ependymoma (9392) | Grade 4 |
|  | Anaplastic oligodendroglioma (9451) | Grade 4 |
|  | Astrocytoma, NOS (9400) | Grade 4 |
|  | Fibrillary astrocytoma (9420) | Grade 4 |
|  | Gemistocytic astrocytoma (9411) | Grade 4 |
|  | Giant cell glioblastoma (9441) | Grade 4, unknown grade |
|  | Glioblastoma, NOS (9440) | Grade 1, Grade 2, Grade 3, Grade 4, unknown grade |
|  | Gliofibroma (9422) | Grade 4, unknown grade |
|  | Gliomatosis cerebri (9381) | Grade 4 |
|  | Malignant glioma (9380) | Grade 4, unknown grade |
|  | Mixed glioma (9382) | Grade 4 |
| Low-grade glioma | Anaplastic astrocytoma (9401) | Grade 2, Grade 3, unknown grade |
|  | Anaplastic oligodendroglioma (9451) | Grade 3 |
|  | Astrocytoma, NOS (9400) | Grade 2, Grade 3, unknown grade |
|  | Fibrillary astrocytoma (9420) | Grade 2, Grade 3, unknown grade |
|  | Gemistocytic astrocytoma (9411) | Grade 2, Grade 3, unknown grade |
|  | Gliomatosis cerebri (9381) | Grade 3 |
|  | Malignant glioma (9380) | Grade 2, Grade 3 |
|  | Mixed glioma (9382) | Grade 2, Grade 3, unknown grade |
|  | Oligodendroglioma, NOS (9450) | Grade 2, Grade 3, unknown grade |
|  | Protoplasmic astrocytoma (9410) | Unknown grade |
| Circumscribed glioma and glioneuronal tumor | Anaplastic astrocytoma (9401) | Grade 1 |
|  | Anaplastic ependymoma (9392) | Grade 3, unknown grade |
|  | Astrocytoma, NOS (9400) | Grade 1 |
|  | Fibrillary astrocytoma (9420) | Grade 1 |
|  | Gliomatosis cerebri (9381) | Grade 1 |
|  | Malignant glioma (9380) | Grade 1 |
|  | Mixed glioma (9382) | Grade 1 |
|  | Oligodendroglioma, NOS (9450) | Grade 1 |
|  | Pleomorphic xanthoastrocytoma (9424) | Grade 1, Grade 2, Grade 3, unknown grade |
|  | Pilocytic astrocytoma (9421) | Grade 1, Grade 2, unknown grade |
|  | Sellar ependymoma (9391) | Grade 1, Grade 2, Grade 3, unknown grade |

Tumor morphology codes and grades were aggregated to create clinically meaningful categories of tumors with common characteristics. Specific groupings are shown in the table and were informed by World Health Organization 2021 classifications.
